# Sickness absence due to otoaudiological diagnoses; a descriptive nationwide study

**DOI:** 10.1186/1471-2458-13-635

**Published:** 2013-07-08

**Authors:** Emilie Friberg, Ulf Rosenhall, Kristina Alexanderson

**Affiliations:** 1 Division of Insurance Medicine, Department of Clinical Neuroscience, Karolinska Institutet, Stockholm, Sweden; 2 Center for Hearing and Communication Research, Department of Clinical Neuroscience, Karolinska Institutet, Stockholm, Sweden; 3 Department of Audiology and Neurotology, Karolinska University Hospital, Stockholm, Sweden

**Keywords:** Otoaudiological diagnoses, Hearing difficulties, Sick leave

## Abstract

**Background:**

Hearing difficulties constitute a large public health problem. Knowledge about their consequences in terms of sickness absence due to otoaudiological diagnoses is very scarce. The aim of this study was to gain such knowledge. Both individuals with sick leave due to otoaudiological diagnoses and sick-leave spells due to these diagnoses were examined, in a nationwide setting.

**Methods:**

Through Swedish nationwide registers we identified all 4768 individuals, aged 16–64 years and living in Sweden who were sickness absent due to otoaudiological diagnoses (ICD10; H60-H95) in 2005. We described the demographic characteristics of these individuals, as well as aspects regarding prevalence and duration of such sick-leave spells, in general and in four specific diagnosis groups; otological, hearing, vertigo, and tinnitus.

**Results:**

Sick leave due to otoaudiological diagnoses was more common among women in all diagnosis groups except with tinnitus. Individuals with a hearing or tinnitus sick-leave diagnosis had a higher educational level and were hospitalized fewer days compared to those sickness absent due to vertigo or otological diagnoses. Particularly, sick-leave spells due to hearing or tinnitus diagnoses tended to be long, in many cases lasting the entire year. The majority of the individuals only had one sick-leave spell in 2005.

**Conclusions:**

Although the actual number of individuals with a sick-leave spell due to specific otoaudiological diagnosis might not be considered high, the high prevalence of long sick-leave spells due to particularly hearing and tinnitus diagnoses indicates the importance of preventive and rehabilitative actions.

## Background

Hearing difficulties constitute a public health problem [1] that is increasing and even estimated to, in 2030, be the ninth leading burden of disease worldwide [2]. Hearing difficulties are associated with communication obstacles and may impact on health related quality of life, and cognitive and emotional status [3-6]. Other ear-related diagnoses emanating from the vestibular part of the inner ear and its central connections are; vertigo, dizziness, and balance disorders. Diseases affecting these systems also cause significant health problems that might lead to sick leave and exclusion from the labor market. Moreover, difficulties to hear is not only a major problem among the elderly - it is also a frequent problem in the working age population, thus potentially having an impact on sick leave [7-9]. Also, it has been suggested that people with hearing difficulties are overrepresented among those on long-term sick leave [10]. Hence, apart from the human suffering, the economic toll might be very high, for individuals, employers, as well as society [3].

In general, the majority of individuals with a disease or impairment, including hearing difficulties, are not sickness absent [11-13], but have hearing aids, other aiding devices, and possibly an adapted work situation. Data on sickness absence is thus seldom a good measure of the occurrence of a disease; however, it is a very good measure of consequences of a disease or impairment, in terms of reduced work capacity [14].

Hearing difficulties may lead to sick leave by way of increased levels of stress. Even a minor hearing difficulty might decrease an individual’s possibilities to be involved in ordinary conversation, especially if several persons are talking at the same time or if there is a lot of background noise, the need to strain to hear might be stressful [7]. Some individuals may even feel stigmatized, and try to hide their difficulty, further increasing the risk of stress and in prolongation potentially also the risk of sick leave. In line with this, a recent study observed that the degree of low-frequency hearing loss was associated with early retirement [15]. Earlier studies have reported that hearing difficulties are more common among men [7,16,17], and also correlated with increased age. Thus analyses of sick leave, stratified by sex and age are warranted.

In spite of the magnitude of the problem, so far only three studies have presented any data describing aspects of individuals on sick leave due to hearing diagnoses [18-20] (one based on the same material as presented here). However, these three studies did not include information regarding the characteristics of the sick-leave spells.

Thus, the objective of this study was to increase the knowledge regarding sick leave due to otoaudiological diagnoses, using different sick-leave measures to give a broad description of both the individuals on sick leave due to these diagnoses and of the characteristics of the sick-leave spells, as well as examine possible differences in this regarding sex and age distributions. This knowledge can be used as a base for initiatives aimed to limit the need for long term sick leave among individuals with otoaudiological problems.

## Methods

Included were all 4768 individuals who lived in Sweden during 2005, who at the start of the year were; aged 16–64, not on disability or early old-age pension, and who in 2005 had had sickness benefits from the Swedish Social Insurance Agency for at least one sick-leave day due to otoaudiological diagnoses (source population 5 102 641 individuals).

For each individual we obtained the following register data:

From Statistics Sweden: information on sex, age, educational level, family situation/constellation, type of living area, country of birth, and old-age pension.

From the Board of Health and Welfare: number of days hospitalized during 2000–2005 (as a measure of prior morbidity).

From the Swedish Social Insurance Agency: data on all benefits for sickness absence (start and end date, diagnoses), disability pensions granted in 2005, and old-age pension.

The unique Personal Identity Number, assigned to all residents in Sweden, was used to link data from the different registers. All individuals in Sweden with income from work or unemployment benefits are covered by the same public sickness insurance, providing sick-leave benefits to people who due to disease or injury cannot work. A sickness certificate issued by a physician is required from the 8th day of a sick-leave spell, the first day is a qualifying day without benefits. Sickness benefits are paid by the Social Insurance Agency, however, the first 14 days of a sick-leave spell is usually paid by the employer. If the work incapacity is prognosticated to be permanent a disability pension can be granted.

Sick-leave spells with ≤6 days in between have been merged as they do not lead to a new qualifying day. Only days with benefits were included in this study. The sick-leave diagnoses were classified according to the International Statistical Classification of Diseases and Related Health Problems, version 10 (ICD-10) [21]. All individuals who had at least one sick-leave spell with an otoaudiological diagnosis as the main diagnose (ICD-10, chapter VIII, H60-H95) in 2005 were included. The otoaudiological diagnoses were categorized as: mainly “otological” (H60-H75, H80, H92, H94, H95), “hearing” (H83, H90, H91), “vertigo” (H81, H82), or “tinnitus” (H93). Individuals with more than one sick-leave spell could be categorized as having sick-leave spells due to several diagnoses.

### Statistical analysis

The following measures of sickness absence were calculated: Frequencies and percentages of: people sickness absent, prevalent and incident sick-leave spells, sick-leave spells/individual, sick-leave spells/duration of sick-leave spells, sick-leave days/individual, sick-leave days/sick-leave spell, and granted disability pension in 2005.

Mean and median values for frequencies were calculated, regarding demographic variables and other characteristics in the different diagnosis groups. Only the sick-leave days compensated in 2005 were included, even if the sick-leave spells had begun before 1 January 2005 or continued after 31 December 2005. The maximum duration of a sick-leave spell was thus 365 days. Sick-leave spells were considered prevalent if the sick leave had begun before 1 January 2005, and otherwise considered incident/new. To examine age and sex differences, the material was stratified by these variables. Statistically significant deviance from equal distribution was assessed with an exact binomial test. Individuals with missing information on any of the variables examined were excluded from that analysis. All statistical tests were two sided, and P-values that were less than 0.05 were considered statistically significant. All statistical analyses were performed using SAS software (version 9.2; SAS Institute, Cary, NC).

### Ethical statement

This project has been evaluated and approved by the Regional Ethical Review Board of Karolinska Institutet, Stockholm, Sweden. The study was based on linkage of several public national registers. Ethical vetting is required when using register data in Sweden. The ethical vetting is performed by regional ethical review boards and the risk appraisal associated with the Law on Public Disclosure and Secrecy is done by data owners. The ethical review boards can, and in relation to this project did, waive the requirement to consult the data subjects directly to obtain their informed consent, this is most often the standard if the research is supported by the ethical review board and the data has already been collected in some other context.

## Results

Demographic characteristics of the 4768 individuals (prevalence 93 per 100 000 individuals in the working population) with sick leave due to otoaudiological diagnoses during 2005, divided by different categories of otoaudiological diagnoses, are presented in Table 1. Sick leave due to all otoaudiological diagnoses was more common among older and female individuals. Sick leave due to vertigo was most common among women (p < 0.001) and sick leave due to tinnitus was slightly more common among men (p < 0.01). Individuals with a hearing or tinnitus sick-leave diagnosis had on average a higher educational level and fewer days hospitalized during 2000–2005 compared to those who had been sickness absent due to vertigo or otological diagnoses. Individuals with sick leave due to otological diagnoses were more often born outside of the European Union.

**Table 1:** **Characteristics** (**frequency and %**) **for all individuals in Sweden with at least one sick**-**leave spell due to otoaudiological diagnoses in 2005**

**Characteristic**		**Sick-leave due to otoaudiological diagnoses**^**a**^				
		**All**	**Otological**^**b**^	**Hearing**^**c**^	**Vertigo**^**d**^	**Tinnitus**^**e**^
Total No.		4768	1326	1012	1476	983
Age groups (years)	16-25	159 (3.33)	85 (6.41)	17 (1.67)	38 (2.57)	20 (2.03)
	26-35	512 (10.74)	245 (18.48)	52 (5.14)	158 (10.70)	59 (6.00)
	36-45	991 (20.78)	328 (24.74)	151 (14.92)	333 (22.56)	183 (18.62)
	46-55	1369 (28.71)	348 (26.24)	295 (29.15)	468 (31.71)	266 (27.06)
	56-65	1737 (36.43)	320 (24.13)	497 (49.11)	479 (32.45)	455 (46.29)
Sex	Women	2781 (58.33)	792 (59.73)	654 (64.62)	909 (61.59)	447 (45.47)
	Men	1987 (41.67)	534 (40.27)	358 (35.38)	567 (38.41)	536 (54.53)
Education (years)	≥12	1738 (35.45)	359 (27.07)	494 (48.81)	456 (30.89)	441 (44.86)
	10-12	2278 (47.78)	744 (56.11)	414 (40.91)	736 (49.86)	396 (40.28)
	0-9	744 (15.60)	220 (16.59)	104 (10.28)	280 (18.97)	145 (14.75)
	Missing	8 (0.17)	-	-	-	-
Family situation	Living with partner without children living at home	1296 (27.18)	248 (18.70)	351 (34.68)	382 (25.88)	321 (32.66)
	Living with partner with children living at home	1757 (36.85)	542 (40.87)	304 (30.04)	604 (40.92)	316 (32.15)
	Living without a partner without children living at home	1340 (28.11)	422 (31.83)	280 (27.67)	359 (24.32)	291 (29.69)
	Living without a partner with children living at home	375 (7.86)	114 (8.60)	77 (7.61)	131 (8.88)	55 (5.60)
Type of living area	Larger cities	1568 (32.89)	498 (37.56)	303 (29.94)	497 (33.67)	283 (28.79)
	Medium sized cities	1829 (38.36)	482 (36.35)	411 (40.61)	562 (38.08)	383 (38.96)
	Smaller places	1371 (28.75)	346 (26.09)	298 (29.45)	417 (28.25)	317 (32.25)
Country of birth	Sweden	4142 (86.87)	1095 (82.58)	907 (89.62)	1278 (86.59)	888 (90.34)
	Other Nordic countries	165 (3.46)	53 (4.00)	37 (3.66)	60 (4.07)	17 (1.73)
	Other European Union countries	102 (2.14)	26 (1.96)	18 (1.78)	32 (2.17)	26 (2.64)
	Rest of the world	359 (7.53)	152 (11.46)	50 (4.94)	106 (7.18)	52 (5.29)
Days hospitalized in 2000-2005	None	2506 (52.56)	493 (37.18)	643 (63.54)	708 (47.97)	676 (68.77)
	<5	1244 (26.09)	495 (37.33)	200 (19.76)	382 (25.88)	175 (17.80)
	≥5	1018 (21.35)	338 (25.49)	169 (16.70)	386 (26.15)	132 (13.43)

^a^ Twenty-nine individuals had more than one sick-leave spell in two different otoaudiological diagnoses groups and thus are included in two categories.

^b^ ICD10: H60-H75, H80, H92, H94, H95.

^c^ ICD10: H83, H90, H91.

^d^ ICD10: H81, H82.

^e^ ICD10: H93.

In the category of otological diagnoses, the most common diagnosis were “suppurative and unspecified otitis media” (ICD-10: H66) responsible for more than a third of the sick-leave spells (Table 2). In the hearing category, the most common diagnosis was “conductive and sensorineural hearing loss” (ICD-10: H90), and “disorders of vestibular function” (ICD-10: H81) was without comparison the most common vertigo diagnosis.

**Table 2:** **Number of sick**-**leave spells due to specific diagnoses within each of the four diagnostic categories used in the analyses**

**Categories**	**Sick-leave diagnoses (ICD-10)**	**Number of sick-leave spells**^**a**^**(%)**
Otological		1358 (100)
	H60 Otitis externa	128 (9.43)
	H61 Other disorders of external ear	29 (2.14)
	H65 Nonsuppurative otitis media	101 (7.44)
	H66 Suppurative and unspecified otitis media	493 (36.30)
	H70 Mastoiditis and related conditions	10 (0.74)
	H71 Cholesteatoma of middle ear	137 (10.09)
	H72 Perforation of tympanic membrane	109 (8.03)
	H74 Other disorders of middle ear and mastoid	43 (3.17)
	H80 Otosclerosis	252 (18.56)
	H92 Otalgia and effusion of ear classified elsewhere	16 (1.18)
	H95 Postprocedural disorders of ear and mastoid process, not elsewhere classified	16 (1.18)
Less than 10 sick-leave spells in 2005, summed	H62 Disorders of external ear in diseases classified elsewhere; H67 Otitis media in diseases classified elsewhere; H68 Eustachian salpingitis and obstruction; H69 Other disorders of eustachian tube; H73 Other disorders of tympanic membrane; H75 Other disorders of middle ear and mastoid in diseases classified elsewhere; H94 Other disorders of ear in diseases	24 (17.67)
Hearing:		1078 (100)
	H83 Other diseases of inner ear	37 (3.43)
	H90 Conductive and sensorineural hearing loss	609 (56.49)
	H91 Other hearing loss	432 (40.07)
Vertigo		1532 (100)
	H81 Disorders of vestibular function	1516 (98.96)
	H82 Vertiginous syndromes in diseases classified elsewhere	16 (1.04)
Tinnitus	H93 Other disorders of ear, not elsewhere classified (including tinnitus and other abnormal auditory perceptions)	1031 (100)

^a^ Total number of spells due to otoaudiological diagnoses: 4999.

Individuals with otological diagnoses had on average fewer sick-leave days compared to individuals with hearing or tinnitus diagnoses. Hearing and tinnitus diagnoses also had a higher proportion of disability pensions granted during the year (Table 3).

**Table 3:** **Different sick**-**leave measures among the individuals with at least one sick**-**leave spell due to otoaudiological diagnoses during 2005**

	**Sick**-**leave diagnoses**				
	**All**	**Otological**	**Hearing**	**Vertigo**	**Tinnitus**
No. of individuals	4768	1326	1012	1476	983
Prevalent sick-leave spells all diagnoses/individual					
Mean/median	1.2/1.0	1.3/1.0	1.2/1.0	1.3/1.0	1.2/1.0
Range	1-11	1-7	1-6	1-11	1-5
Incident sick-leave spells all diagnoses/individual					
Mean/median	0.8/1.0	1.1/1.0	0.6/1.0	0.9/1.0	0.6/0.0
Range	0-10	0-6	0-6	0-10	0-5
No. of sick-leave spells all diagnoses/individual					
1	3912	1046	859	1181	826
2	709	227	135	240	130
>2	147	53	18	55	27
No. of sick-leave days all diagnoses/individual	122.6/59.0	45.0/14.0	178.4/151.0	107.6/45.0	193.8/181.0
Mean/median					
No. of sick-leave days due to otoaudiological diagnoses/individual					
1-27	1866	973	160	620	124
28-180	1386	180	388	467	368
181-364	728	46	258	167	253
365	610	42	185	161	222
No. of DP 2005	546	25	221	95	207

*DP,* Disability pension.

Together, the 4768 individuals had 4999 prevalent sick-leave spells due to otoaudiological diagnoses during the year 2005 (Table 4). The median length of a sick-leave spell due to otological diagnoses was 11 days, in contrast to sick-leave spells due to tinnitus (150 days) and hearing diagnoses (124 days) where the median length of the sick-leave spells was considerably longer (Table 4).

**Table 4:** **Distribution and characteristics of sick**-**leave spells due to otoaudiological diagnoses in 2005**

	**At least one sick**-**leave day due to otoaudiological diagnoses**				
	**All**	**Otological**	**Hearing**	**Vertigo**	**Tinnitus**
No. of prevalent sick-leave spells	4999	1358	1078	1532	1031
No. of incident sick-leave spells	3313	1205	544	1089	475
No. of sick-leave days/spell	112.0/41.0	35.9/11.0	162.8/124.0	97.3/33.0	178.7/150.0
Mean/median					
No. of sick-leave spells with different durations					
1-27	2018	1016	198	653	151
28-180	1492	175	423	498	398
181-364	698	46	248	160	244
365	610	42	185	161	222

Note: sick-leave spells with ≤6 days in between have been merged.

The analyses stratified by sex revealed only minor differences between women and men (Table 5). In the analyses stratified by age we observed that individuals 50 years old or older had more sick-leave days during the year and were more often granted a disability pension than individuals younger than 50 (Table 5).

**Table 5:** **Description of sick leave among individuals with at least one sick**-**leave day due to otoaudiological diagnoses in 2005**, **stratified by sex and age**

	**Otoaudiological diagnoses**				
	**All**	**Otological**	**Hearing**	**Vertigo**	**Tinnitus**
Women					
No. of individuals	2781	792	654	909	447
Prevalent sick-leave spells all diagnoses/individual					
Mean/median	1.2/1.0	1.3/1.0	1.2/1.0	1.3/1.0	1.2/1.0
Range	1-7	1-7	1-6	1-5	1-5
No. of sick-leave days all diagnoses/individual	122.5/59.0	49.5/14.0	182.3/151.0	109.8/47.0	192.0/163.0
Mean/median					
No. of individuals with sick-leave spells of different durations					
1-27	1090	563	95	377	56
28-180	816	105	265	284	175
181-364	394	32	158	108	103
365	366	33	128	98	107
No. of DP 2005	307	17	149	62	81
Men					
No. of individuals	1987	534	358	567	536
Prevalent sick-leave spells all diagnoses/individual					
Mean/median	1.2/1.0	1.2/1.0	1.3/1.0	1.2/1.0	1.2/1.0
Range	1-11	1-5	1-4	1-11	1-5
No. of sick-leave days all diagnoses/individual					
Mean/median	122.7/59.0	38.4/14.0	171.4/151.0	104.0/42.0	195.3/181.0
No. of individuals with sick-leave spells of different durations					
1-27	776	405	63	241	68
28-180	570	77	124	182	190
181-364	334	17	104	62	155
365	244	9	57	63	115
No. of DP 2005	239	8	72	33	126
Age <50 years^a^					
No. of individuals	2125	783	306	705	340
Prevalent sick-leave spells all diagnoses/individual					
Mean/median	1.2/1.0	1.3/1.0	1.2/1.0	1.3/1.0	1.2/1.0
Range	1-5	1-5	1-4	1-5	1-5
No. of sick-leave days all diagnoses/individual					
Mean/median	96.7/31.0	40.2/13.0	154.6/120.0	96.3/35.0	177.5/150.0
No. of individuals with sick-leave spells of different durations					
1-27	1048	593	78	326	52
28-180	530	94	107	207	126
181-364	236	25	68	67	80
365	208	19	47	69	73
No. of DP 2005	125	9	47	29	40
Age ≥50 years^a^					
No. of individuals	2643	543	706	771	643
Prevalent sick-leave spells all diagnoses/individual					
Mean/median	1.2/1.0	1.3/1.0	1.2/1.0	1.2/1.0	1.2/1.0
Range	1-11	1-7	1-6	1-11	1-5
No. of sick-leave days all diagnoses/individual					
Mean/median	143.3/90.0	52.0/15.0	188.8/167.0	117.8/57.0	202.4/194.0
No. of individuals with sick-leave spells of different durations					
1-27	818	375	80	292	72
28-180	856	88	282	259	239
181-364	492	24	194	103	178
365	402	23	138	92	149
No. of DP 2005	421	16	174	66	167

Note: sick-leave spells with ≤6 days in between have been merged. *DP,* Disability pension.

^a^ Mean age “all” 49.

**Figure 1:**
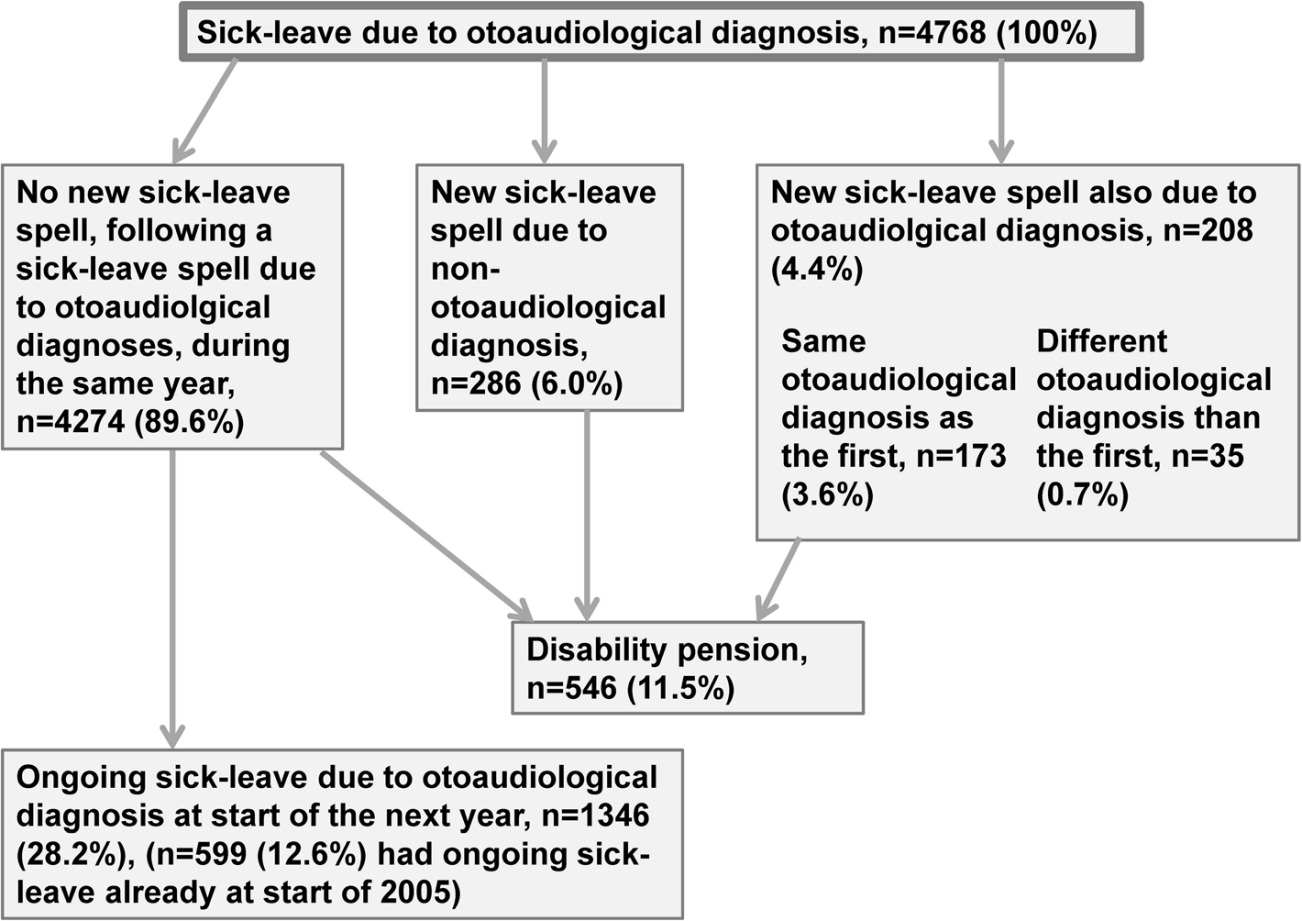
Distributions (frequency and (percentage of total number of individuals)) of sickness absence or disability pension in 2005, among those having a sick-leave spell due to otoaudiological diagnosis in 2005.

The most common scenario (90%) following a sick-leave spell due to otoaudiological diagnoses was that there was no new sick-leave spell during the same year, this could be because the first sick-leave spell was still ongoing, because of the individual being granted disability pension, or because of return to work/unemployment (Figure 1). Six percent of the individuals had a new sick-leave spell due to a non-otoaudiological diagnosis and four percent had another sick-leave spell due to otoaudiological diagnoses, 83% due to the same diagnosis as the first one. Of the 4768 individuals with sick leave due to otoaudiological diagnoses in 2005, 12% were granted a disability pension during the same year (Figure 1).

## Discussion

This population-based study describes all individuals aged 16–64 years who in 2005 lived in Sweden and were not on old-age or disability pension in the beginning of the year, and who had at least one sick-leave spell due to otoaudiological diagnoses in 2005. Sick-leave due to otoaudiological diagnoses was more common among women in all diagnosis groups, with the exception of tinnitus, where we observed a slight majority of men. The higher prevalence of sick leave related to hearing among women than men (65% compared to 35%) is an important finding. In epidemiological literature a well-known observation is that men have poorer hearing than women among adults [16,17]. Also, men are more often exposed to occupational noise than women. Diagnoses related to vertigo are however generally observed to be more prevalent among women than men [22]. Our results indicate that the social consequences in terms of sickness absence seem to be higher for women. There are several possible explanations for this; women may for example depend on hearing in their work to a higher extent and thus be more vulnerable to a decrease in hearing capacity. Other types of studies are needed to investigate why sick leave due to hearing difficulties is more common among women.

The treatment of chronic middle ear diseases, like chronic otitis media, cholesteatoma, and otosclerosis, has improved dramatically due to the introduction of antibiotics and modern middle ear surgery, compared to the situation in the mid 20th century. However, chronic middle ear diseases still imply health problems that are clearly discernible in this study by the presence of patients, most often women and individuals born in a non European country, with sick-leave spells related to these diagnoses.

We found that particularly sick-leave spells due to hearing or tinnitus diagnoses tended to be long; in many cases they lasted the entire year. However, the vast majority of individuals only had one sick-leave spell during the year.

We have recently published a study of the risk of disability pension among these individuals with sick leave due to otoaudiological diagnoses, where we found a threefold risk of disability pension among those on sick leave due to audiological diagnoses (hearing and tinnitus) compared to those with sick leave due to non-otoaudiological diagnoses [20]. Only a few prior studies have presented characteristics of individuals on sick leave due to specific or any hearing diagnoses [18-20] (one based on the same data material [20]), overall showing results in agreement with ours. To the best of our knowledge, this nationwide study describing sick leave due to otoaudiological diagnoses is the first to present information regarding the characteristics of such sick-leave spells.

### Strengths and limitations

Major strengths of this study include its population-based nationwide design (not a sample), comprising all individuals in Sweden with a sick-leave spell due to otoaudiological diagnoses during one year. Other strengths are the high quality of the population-based registers and that the information was not self-reported [23,24]. The wide range of demographic variables available is also an advantage as is the detailed information regarding sick-leave diagnoses. Such information is seldom available in large population-based studies, and made it possible to present several commonly used measures of sick leave, which facilitate comparison with future studies. The validity of sick-leave diagnoses is often discussed, however, seldom studied. We know of only one such study, which reported good validity of sick-leave diagnoses, when comparing them to the diagnoses stated in the medical records of the patients [25].

One limitation is that only information about the main diagnosis legitimizing the sickness absence was available. This means that for some sick-leave spells, hearing diagnoses might for example have been mentioned as a contributing diagnosis, thus leading to an underestimation of sickness absence related to hearing difficulties. Another limitation is that for employed individuals, information about most sick-leave spells shorter than 15 days were not included, thus the frequency of sick-leave spells observed in this study is an underestimation of the true number.

## Conclusion

This nationwide population-based descriptive study presents new information of the characteristics of individuals with sick leave due to otoaudiological diagnoses as well as new information on their sick-leave spells. We observed a high prevalence of long and very long sick-leave spells due to these diagnoses which stress the great importance of preventive and work adaptive measures among individuals with hearing difficulties to minimize the need for long-term sick leave and permanent exclusion from the labor market.

## Competing interests

The authors declared that they have no competing interest.

## Author’s contributions

Study concept and design: EF, KA. Acquisition of data: KA. Analysis and interpretation of data: EF, UR, KA. Drafting of the manuscript: EF. Critical revision of the manuscript for important intellectual content: UR, KA. Administrative, technical or material support: KA. All authors have read and approved the final manuscript.

## Pre-publication history

The pre-publication history for this paper can be accessed here:

http://www.biomedcentral.com/1471-2458/13/635/prepub
